# Intravascular papillary endothelial hyperplasia (Masson's tumour) in the jejunum: A case report of unusual gastrointestinal bleeding.

**DOI:** 10.1016/j.ijscr.2024.109501

**Published:** 2024-03-11

**Authors:** Marcelis Jordan, Carminati Alessia, Koessler Thibaud, Toso Christian, Ris Frederic, Sleiman Marwan

**Affiliations:** a University Hospitals of Geneva, Division of Digestive Surgery, Geneva, Switzerland; b University Hospitals of Geneva, Division of Pathology, Geneva, Switzerland; c University Hospitals of Geneva, Division of Oncology, Geneva, Switzerland

**Keywords:** Gastrointestinal bleeding, Masson's tumour, Intravascular papillary endothelial hyperplasia

## Abstract

**Introduction:**

Intravascular papillary endothelial hyperplasia (IPEH), originally described by Pierre Masson in 1923, is a benign vascular lesion characterised by the reactive proliferation of endothelial cells. This tumour typically manifests on the fingers, head, neck, or trunk. However, involvement of other organ systems, including abdominal organs, is possible although exceedingly rare.

**Case presentation:**

A 57-year-old male patient presented to the emergency department with a 24-h history of recurrent light-headedness. The patient was haemodynamically stable, and physical examination showed no abdominal pain. Digital rectal examination unveiled melena. Initial blood analysis indicated a haemoglobin level of 10.5 g/dL. Comprehensive workup with abdominal computed tomography, upper and lower endoscopy, and gastrointestinal lumen MRI failed to yield significant findings.

**Discussion:**

On the fourth day of hospitalisation, persistent melena and transfusion of four units of blood triggered a new upper endoscopy. This endoscopy extended to the jejunum, revealing a submucosal lesion measuring 20 mm, situated approximately 40 cm distal to the ligament of Treitz. Due to ongoing intraluminal bleeding with decreased haemoglobin levels, a segmental jejunal resection was performed. Pathological examination confirmed the diagnosis of a completely resected submucosal IPEH.

**Conclusion:**

IPEH, also known as Masson's tumour, is an uncommon vascular lesion within the intestinal tract, occasionally resulting in persistent haemorrhage. The preferred treatment is total surgical resection, with a low likelihood of recurrence. Currently, postoperative surveillance is not recommended. To the best of our knowledge, no cases of recurrence have been documented for Masson's tumour localised in the gastrointestinal tract in the existing literature.

## Highlights

• Intravascular Papillary Endothelial Hyperplasia (IPEH), or Masson Tumor, is a benign vascular lesion.
• This benign tumor rarely involve the gastrointestinal lumen: eight cases of IPEH reported.
• IPEH can be responsible for bleeding,it should be considered after ruling out other causesbeforetotal surgical resection for treatment.
• Recurrence of IPEH is extremely rare; that’s why no follow-up is currently recommended.

## 1 Introduction

Intravascular papillary endothelial hyperplasia (IPEH), first described in 1923 by Pierre Masson, is a benign vascular lesion characterised by reactive proliferation of endothelial cells [[1], [2], [3]]. This benign tumour most frequently occurs on the fingers, head and neck, or trunk. Furthermore, these lesions can develop within any blood vessel and rarely involve other systems, such as abdominal organs, with only a few cases reported [3,6,[8], [9], [10], [11], [12], [13], [14]]. In this report, we present a case of IPEH responsible for persistent bleeding localised in the jejunum. This report was drafted in line with the SCARE 2023 criteria [15].

## 2 Case presentation

A 57-year-old male patient with overweight (weight 97 kg, height 186 cm, BMI 28 kg/m^2^) presented to the emergency department at University Hospital with a 24-h history of multiple episodes of light-headedness. His medical history included ischemic heart disease treated with stent placement in 2017 and long-term aspirin use without any previous abdominal surgery. The patient was haemodynamically stable, and physical examination showed no abdominal pain. Digital rectal examination unveiled melena. Blood analysis showed a haemoglobin level of 10.5 g/dL, leukocytes of 8.2 × 10^3^/μL, and C-reactive protein of 13.92 mg/L; finally, arterial lactate levels were measured at 3.0 mmol/L. During his stay in the emergency unit, the patient passed two melanic stools, and his haemoglobin levels dropped to 8.6 g/dL. A first upper endoscopy showed no active bleeding and no lesion.

**Fig. 1:**
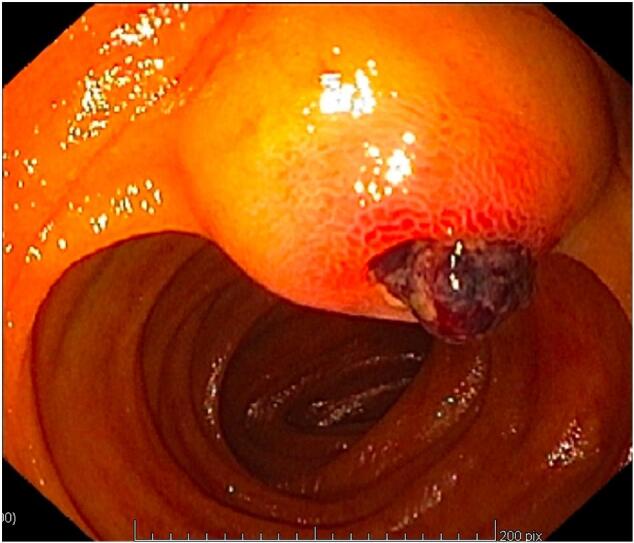
Upper gastrointestinal endoscopy findings. A 20-mm submucosal lesion located approximately 40 cm distally to the ligament of Treitz.

The patient was admitted to the middle care unit, where he experienced haemodynamic instability and a further drop in haemoglobin levels to 7.0 g/dL. Abdominal CT did not reveal any active bleeding or suspicious gastrointestinal neoplasm. A second diagnostic gastroscopy and a colonoscopy were also carried out but yielded no significant findings. On the fourth day, due to persistent melena and the transfusion of four units of blood since admission, an upper endoscopy was repeated. This endoscopy extended to the jejunum and revealed a submucosal lesion measuring 20 mm, located approximately 40 cm distally to the ligament of Treitz, and a fresh blood clot was observed (Fig. 1). An MRI of the gastrointestinal lumen did not contribute to the diagnosis. Given the clinical findings, the decision was made to proceed with an exploratory laparoscopy. During the procedure, the segment of the jejunum containing the observed mass was resected, followed by a side-to-side peristaltic anastomosis. The patient was discharged on the sixth day postoperatively without any complications.

**Fig. 2:**
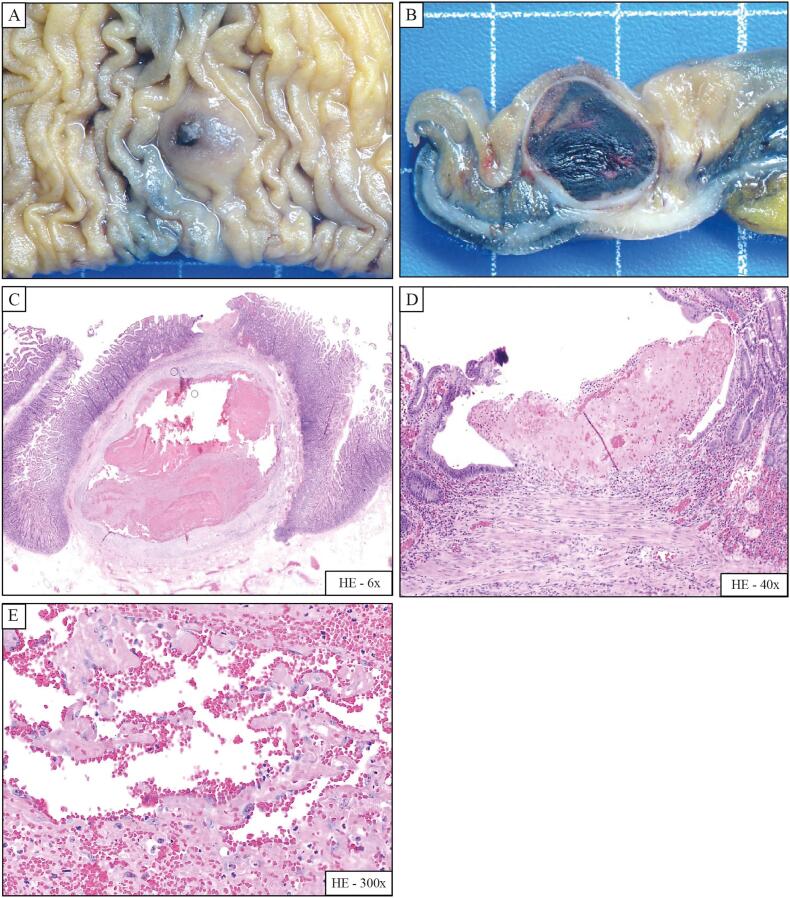
Pathological analysis of the jejunectomy specimen. Gross analysis confirmed a submucosal tumour measuring 0.8 × 0.8 cm, with a millimetre-sized ulcer (A). The cut surface showed a whitish capsule with bloody content, limited to the submucosa layer (B). Histological examination revealed a vascular structure partially thrombosed and repermeabilised (haematoxylin-eosin, 6×; C), with an ulceration of the mucosa above the tumour (haematoxylin-eosin, 40×; D), and intravascular papillary endothelial hyperplasia (haematoxylin-eosin, 300×; E).

The gross analysis of the jejunectomy specimen showed a central stenosis. After opening the jejunal segment, a rounded purplish protuberance of the mucosa measuring 0.8 × 0.8 cm was found, centred by a millimetre-sized ulcer (Fig. 2A). On cross-section after formalin fixation, the tumour presented a cut surface made of a whitish capsule with bloody content, measuring 1 × 0.8 × 0.8 cm. The tumour was intramural, limited to the submucosa layer (Fig. 2B). No other macroscopic lesion was seen.

Histological analysis revealed a large vascular structure in the superficial submucosal layer, partially thrombosed and repermeabilised (Fig. 2C). An ulceration of the mucosa above the tumour was seen (Fig. 2D). Numerous papillary structures with cores of fibrin and lined by a single layer of plump endothelial cells were seen (Fig. 2E). There were no significant atypia, no mitotic figures, and no necrosis. A final diagnosis of completely resected submucosal papillary endothelial hyperplasia associated with a mucosal ulcer was retained. No additional treatment or follow-up was indicated for this benign tumour in a rare location.

## 3 Discussion

Intravascular papillary endothelial hyperplasia (IPEH) is histologically characterised by the proliferation of endothelial cells in a papillary formation, frequently associated with a thrombus within the vascular lumen. This entity was initially hypothesised to be a neoplasm and was named ‘hémangioendothéliome végétant intravasculaire’. It was first described by Pierre Masson in the lumen of haemorrhoid veins in 1923 [1]. However, in 1932, Henschen clarified that the disease is reactive in nature, with endothelialisation occurring around fragments of thrombi, rather than being a true neoplasm [4]. Finally, to better reflect its pathophysiology, the entity was termed ‘intravascular papillary endothelial hyperplasia’ in 1976 by Clearkin and Enzinger [5]. IPEH is categorised into three types: the pure form (55.8 %) occurring de novo in a dilated vascular space with no causative aetiology; the mixed form (39.9 %), a superimposed lesion over a pre-existing vascular abnormality (arteriovenous malformation or haemangioma); and the extravascular form (4.3 %) occurring with endothelial proliferation at a hematoma after trauma [6,7]. The exact origin of this benign lesion remains unclear and continues to be the subject of numerous immunohistochemistry studies.

IPEH lesions are exceedingly rare within the gastrointestinal tract, with only a few reports in the literature. Their non-specific clinical presentation often poses challenges in diagnosis. To date, eight cases of IPEH within the gastrointestinal lumen have been reported: one in the duodenum, five in the jejunum, one in the ileum, and one in the cecum [3,[8], [9], [10], [11], [12], [13], [14]].

There are no specific imaging appearances for IPEH on CT, MRI, or angiographic patterns, making differential diagnosis challenging. Depending on the affected site, clinical features of IPEH may mimic other benign lesions (such as haemangiomas or pyogenic granulomas), infectious conditions (like bacillary angiomatosis or Kaposi's sarcoma), or even malignant tumours (including angiosarcoma).

A review of the literature, along with these presented cases, revealed that confusion with other clinical entities is common. A definitive diagnosis of IPEH is often not reached during preoperative examinations but is confirmed through histomorphological examination after pathology analysis. Histomorphological examination is crucial to differentiate benign IPEH lesions from malignant tumours capable of metastasis, such as angiosarcoma. The criteria for distinguishing IPEH lesions from malignant angiosarcoma include an intraluminal lesion origin, minimal necrosis, a close association with organised thrombus, and the absence of pleomorphic and mitotic activity in cells [4,16,17]. The demonstration of vascular origin and the proliferative index through immunohistochemistry can significantly contribute to the accurate differential diagnosis of IPEH. Essential antigens for this purpose include CD31 and CD34, which, while not endothelial cell-specific, are widely expressed by vascular endothelium, particularly under pathological conditions [2].

The treatment of choice for IPEH is total surgical resection, and recurrence is extremely rare when achieving R0 margins, regardless of the lesion's location. Recurrences have been reported in mixed and extravascular varieties in series of skin cases [1,6]. No specific surveillance is recommended, primarily due to the benign nature of the lesion and its low recurrence rate. To the best of our knowledge, no cases of recurrence have been reported for Masson's tumour with gastrointestinal localisation in the literature.

## 4 Conclusion

Intravascular papillary endothelial hyperplasia, also known as Masson's tumour, is a benign vascular lesion that rarely occurs within the intestinal tract. The prevalence of this entity in the gastrointestinal tract remains unknown due to the limited number of reported cases. In the event of gastrointestinal bleeding without an evident cause identified through investigative procedures, it is crucial not to overlook this rare condition as a potential differential diagnosis, which may prompt earlier surgical intervention. The definitive diagnostic method for IPEH continues to be pathological examination of the specimen. The optimal treatment for this cause of gastrointestinal bleeding is surgical resection, with a low recurrence rate observed in cases of the pure form, as seen in our presented case. Therefore, no follow-up is currently recommended.

Written informed consent was obtained from the patient for publication of this case report and accompanying images. A copy of the written consent is available for review from the Editor-in-Chief of this journal on request.

## Ethical approval

No ethics committee approval is required for the submission of this case to a scientific journal at the University of Geneva and HUG.

## Funding

None.

## Guarantor

Dr. Med. Jordan Marcelis.

## Research registration number

No study series.

## CRediT authorship contribution statement

**Marcelis Jordan:** Conception and design of the article, acquisition of data and interpretation of data. Drafting the article.
**Carminati Alessia:** Acquisition of data and interpretation of data.
**Koessler Thibaud:** Article initiative and revising the article critically for important intellectual content. Final approval of the version to be submitted.
**Toso Christian:** Revising the article critically for important intellectual content.
**Ris Frederic:** Revising the article critically for important intellectual content.
**Sleiman Marwan:** Supervision and revising the article critically for important intellectual content. Final approval of the version to be submitted.

## Declaration of competing interest

None.
